# Dynamic time warping assessment of high-resolution melt curves provides a robust metric for fungal identification

**DOI:** 10.1371/journal.pone.0173320

**Published:** 2017-03-06

**Authors:** Sha Lu, Gordana Mirchevska, Sayali S. Phatak, Dongmei Li, Janos Luka, Richard A. Calderone, William A. Fonzi

**Affiliations:** 1 Dermatology Department, Sun Yat-sen Memorial Hospital, Sun Yat-sen University, Guangzhou, China; 2 Institute for Microbiology and Parasitology, University Sts Cyril and Methodius, Skopje, Macedonia; 3 Department of Microbiology & Immunology, Georgetown University, Washington, DC, United States of America; 4 Walter Reed Army Institute of Research (WRAIR), Silver Spring, MD, United States of America; Institute of Microbiology, SWITZERLAND

## Abstract

Fungal infections are a global problem imposing considerable disease burden. One of the unmet needs in addressing these infections is rapid, sensitive diagnostics. A promising molecular diagnostic approach is high-resolution melt analysis (HRM). However, there has been little effort in leveraging HRM data for automated, objective identification of fungal species. The purpose of these studies was to assess the utility of distance methods developed for comparison of time series data to classify HRM curves as a means of fungal species identification. Dynamic time warping (DTW), first introduced in the context of speech recognition to identify temporal distortion of similar sounds, is an elastic distance measure that has been successfully applied to a wide range of time series data. Comparison of HRM curves of the rDNA internal transcribed spacer (ITS) region from 51 strains of 18 fungal species using DTW distances allowed accurate classification and clustering of all 51 strains. The utility of DTW distances for species identification was demonstrated by matching HRM curves from 243 previously identified clinical isolates against a database of curves from standard reference strains. The results revealed a number of prior misclassifications, discriminated species that are not resolved by routine phenotypic tests, and accurately identified all 243 test strains. In addition to DTW, several other distance functions, Edit Distance on Real sequence (EDR) and Shape-based Distance (SBD), showed promise. It is concluded that DTW-based distances provide a useful metric for the automated identification of fungi based on HRM curves of the ITS region and that this provides the foundation for a robust and automatable method applicable to the clinical setting.

## Introduction

Fungal infections are a global problem causing considerable disease burden [1]. One of the unmet needs in addressing these infections is rapid, sensitive diagnostics [1]. A promising molecular diagnostic approach is high resolution melt analysis (HRM), a method pioneered by Wittwer and colleagues [2, 3]. The method is elegant in its simplicity and sensitivity. Following PCR amplification of a target DNA in the presence of an intercalating fluorescent dye, controlled heating of the sample results in gradual melting of the DNA. The transition of the DNA from double to single stranded and the resulting dissociation of the intercalating fluorescent dye is reflected in the associated decline in fluorescence [2, 3]. Because the melting process is a function of the DNA sequence and the relative position of cooperative melting domains [4], as little as a single nucleotide difference can alter the melting curve [2, 3].

HRM analysis of various amplicons has been applied to the identification of fungal species. Multiple studies have employed the rDNA internal transcribed spacer (ITS) region, either in part, or in its entirety, as the target amplicon. Dunyach et al. [5] used universal primers to amplify the ITS1-5.8S-ITS2 region and showed that *C*. *albicans*, *C*. *tropicalis*, *C*. *parapsilosis*, *C*. *glabrata*, and *C*. *krusei* (Pichia kudriavzevii) could be distinguished by HRM analysis. Similarly, Alnuaimi et al. [6] showed that HRM curves of the ITS1-5.8S-ITS2 region could discriminate between eight species of *Candida*. Other studies have analyzed HRM curves of the ITS2 region and successfully resolved as many as 23 different *Candida* species [7–9]. Cryptococcal species were delineated based on the melt profile of the ITS1 region [10], while a variable region of the 18S rDNA allowed differentiation of six species of *Mucorales* [11]. Melt profiles of the rDNA intergenic spacer region readily distinguished cryptic species within the *C*. *parapsilosis* spp. complex [12].

While most studies have focused on the rDNA region, other amplicons have also been examined. The melt profile of the gene encoding mannoprotein MP65 can discriminate 5 species of *Candida* [13] and Plachy et al. studied the melt curves of random amplified polymorphic DNA, a technique designated McRAPD [14]. The utility of HRM profiles in strain typing was shown with the amplified products of *CDC3*, *EF3*, and *HIS3* [15, 16].

In these studies HRM data was assessed using two methods, melting point (T_M_) determination and difference curve analysis. Tm determination provides the simplest assessment. Although this single point parameter is sufficient to discriminate a limited number of species [5, 11], it lacks the resolution needed to distinguish all the species of clinical interest [7–9]. Most studies have relied on visual assessment of difference curves, alone or in combination with T_M_ determinations [6–10, 12]. Difference curves are generated by subtraction of a standard curve from the test curves after normalization and temperature shifting of the raw melt curve. Temperature shifting accounts for well-to-well differences by aligning all the curves that are presumed should be the same. Visual assessment of the difference curves easily discriminates most all species that have been examined [6–10, 12]. Despite its discriminatory power, the time and labor costs and subjectivity of visual assessment prevent its adoption in clinical settings where an objective, scalable, and automatable approach is needed.

Two studies in the fungal literature have explored computational methods of evaluating curve similarities. In the study by Mandviwala et al. [17] the authors constructed a distance matrix of Manhattan distances between melt rate curves and showed by minimum match scoring that eight species of *Candida* could be distinguished. Trtkova el al. [18] took a similar approach examining the Manhattan distances between normalized melt curves of RAPD results. However, this proved inferior to comparison of difference curves [18].

The sequentially ordered data of a melt curve is comparable to the temporally ordered data of a time series. The analysis of time series data has importance in a broad range of applications from speech recognition to stock price fluctuations to astronomical observations [19, 20]. One of the more successful distance measures in this domain is dynamic time warping (DTW), first introduced in the context of speech recognition to identify temporal distortion of similar sounds [21]. In this study we examine the application of DTW to the classification of HRM curves of the ITS region of 18 species of fungi and demonstrate its ability to correctly cluster and classify all of the species tested including several that are difficult to differentiate by traditional phenotypic methods. As a practical test of its utility, DTW distances were used in a minimum score classification scheme to correctly identify 243 clinical isolates. The potential utility of several other time series functions was also examined. These results lay the foundation for a rapid, objective, and automatable method for the identification of clinically relevant fungal species.

## Materials and methods

### Fungal strains

Standard strains and their sources are listed in Table 1. Clinical isolates were taken from a previously described collection of oral and vaginal yeast cultured from HIV positive and HIV negative women [22].

**Table 1:** Strains used in the present study.

Species	Strain	Source/Reference	GenBank Accession No.^f^
*Aspergillus flavus*	No.1	CAMS ^a^	
	gl-5	CAMS	
*Aspergillus fumigatus*	SUMS_0672	SYMH ^b^	KT067746
	8–21	CAMS	KT067747
	10–119	CAMS	KT067748
*Aspergillus terreus*	SUMS_0191	SYMH	
	SUMS_0769	SYMH	KT067754
*Candida albicans*	SC5314	Squib Culture Collection	
	CI_129v	[22]	KT067740
	CI_143o	[22]	KT067743
	CI_193o	[22]	KT067744
	CI_238o	[22]	KT067742
*Candida dubliniensis*	Y-17512	ARS(NRRL)^c^	KY514056
	Y-27787	ARS(NRRL)	KY514057
*Candida glabrata*	Y-65	ARS(NRRL)	
	CI_36o	[22]	KT067758
	CI_100v	[22]	KT067759
*Candida orthopsilosis*	Y-48468	ARS(NRRL)	
	Y-27060	ARS(NRRL)	
*Candida parapsilosis*	Y-12969	ARS(NRRL)	
	Y-182	ARS(NRRL)	
*Candida tropicalis*	Y-607	ARS(NRRL)	
	Y-48158	ARS(NRRL)	
	CI_155v	[22]	KT067761
	CI_243o	[22]	KT067762
*Candida zeylanoides*	Y-1403	ARS(NRRL)	
	Y-1774	ARS(NRRL)	
*Clavispora lusitaniae*	Y-11827	ARS(NRRL)	
	Y-48268	ARS(NRRL)	
*Cryptococcus gattii*	ATCC MYA4560	MWHC ^d^	
	R1396	June Kwon-Chung	
	W14-276	June Kwon-Chung	
*Cryptococcus neoformans*	JEC-21	YGSC^e^	
	H99	YGSC	
	SUMS_0043	SYMH	AB436637.1
	SUMS_0044	SYMH	AB436638.1
	SUMS_0046	SYMH	AB436640.1
*Debaryomyces hansenii*	Y-1448	ARS(NRRL)	
	Y-1449	ARS(NRRL)	
*Myerozyma guilliermondii*	Y-2076	ARS(NRRL)	
	Y-27821	ARS(NRRL)	
	ATCC-6260	MWHC	
*Pichia kudriavzevii*	ATCC-6258	MWHC	
	Y-27803	ARS(NRRL)	
	Y-27825	ARS(NRRL)	
*Rhodotorula mucilaginosa*	Y-1591	ARS(NRRL)	
	Y-2510	ARS(NRRL)	
*Talaromyces marneffei*	SUMS_0429	SYMH	KT067767
	SUMS_0556	SYMH	JN679223.1
	SUMS_0624	SYMH	JQ912272.1
	SUMS_0751	SYMH	KT121405

a. Chinese Academy of Medical Science

b. Sun Yat-Sen Memorial Hospital

c. Agricultural Research Service (Northern Regional Research Laboratory)

d. Medstar Washington Hospital Center

e. Yeast Genetics Stock Center

f. Species identity was verified by partial sequencing of the ITS or 25S region of rDNA

### DNA preparation

Yeast strains were cultured in 2 ml YPD medium (1% yeast extract, 2% Bacto Peptone, 2% glucose) for approximately 24 h at 28°C, molds were cultured for 48–72 h. Yeast cells were collected by centrifugation, mycelia were collected on Spin-X centrifuge tube filters (Corning Costar). After washing with 1 ml H_2_O, cells were suspended in 1 ml of lysis buffer (500 mM NaCl, 50 mM Tris-HCl, pH 8.0, 50 mM EDTA, and 4% SDS) and processed according to the “repeated bead beating plus column” method of Yu and Morrison [23] except that an Isolate II Genomic DNA Kit (Bioline) was used to purify the DNA. The concentration of purified DNA was determined using a NanoDrop spectrophotometer. The purified DNA samples were used to characterize species specificity of HRM curves and for testing and optimizing distance functions.

To facilitate throughput, melt curves of clinical isolates and standard yeast strains were obtained using a crude DNA preparation [8]. Isolates were inoculated into 150 μl of YPD in 96-well plates and incubated 24 h at 28°C. Cells were collected by centrifugation, washed twice with 100 μl of 10 mM Tris-HCl, pH 8.5, and suspended in 100 μl of Tris buffer. Samples were heated to 95°C for 15 min, frozen at -80°C for 15 min, and thawed at room temperature [8]. The thawed samples were centrifuged and the DNA containing supernatant was recovered. DNA samples were stored at -20°C.

### PCR amplification

ITS amplicons were PCR amplified as described by Toju et al. [24]. ITS1 was amplified using primers ITS1-F_KYO2 (TAGAGGAAGTAAAAGTCGTAA) and ITS2_KYO2 (TTYRCTRCGTTCTTCATC). ITS2 was amplified using primers ITS3_KYO2 (GATGAAGAACGYAGYRAA) and ITS4 (TCCTCCGCTTATTGATATGC). The complete ITS1-5.8S-ITS2 region was amplified with primers ITS1-F_KYO2 and ITS4. PAGE purified primers were purchased from Integrated DNA Technologies. The PCR reaction consisted of 1X SensiFAST HRM mix (Bioline), 0.5 μM of each primer and 1 ng of purified DNA template or 2 μl of freeze-thaw DNA preparation in a total volume of 20 μl. Amplification was performed in a LightCycler 480 (Roche) using the following thermal cycles: 1 cycle of 95°C for 5 min, followed by 40 cycles of 95°C for 10 s, 47°C for 10 s and 72°C for 12 s.

A high-resolution melt profile was acquired following amplification. The samples were heated to 95°C for 1 min and cooled to 40°C for 1 min to allow strand hybridization. Samples were then heated from 60–95°C at a rate of 0.02°C/s with acquisition of 25 fluorescent readings per 1°C. All samples were tested in duplicate in two independent trials. A negative control lacking DNA was included. Samples were subsequently analyzed by agarose gel electrophoresis to verify the presence of a single product of the predicted size.

### High-resolution melt curve analysis

HRM produces normalized melt curves, which are all similar in that the relative fluorescence of the sample ranges from 100% to 0%. The curves differ in the rate at which fluorescence declines with increasing temperature. The commonly used difference curve captures the relative difference in fluorescence between two melt curves, an indirect measure of differences in melt rate. Sample classification requires that all samples be assessed against a consistent reference curve for comparison. For these studies, rather than an indirect, relative measure, melting rates were directly determined using the first derivative of the normalized melt curves.

Melt curve data was normalized within the LightCycler 480 Gene Scanning Software (Roche). The region between 72 and 74°C was set at 100%, 93.5 to 94°C was set to 0%. No curve shifting was applied. All subsequent data processing was performed in R [25]. Melting rates were calculated from the negative first derivative of the melt curve. Rate curves were fitted with a cubic smoothing spline using the default leave-one-out cross-validation of the splines package and the splines were used to calculate rates at 0.1°C increments. The resulting curves were z-normalized using the scale function and the region between 76 and 94°C was used for distance calculations.

Dynamic time warping (DTW) distances were calculated using the DTW package of Giorgino [19] within a call to “proxy” [26]. Implementations of the distance functions Edit Distance on Real sequence (EDR) [27], Edit distance with Real Penalty (ERP) [28], and Longest Common Subsequences (LCSS) [29] were provided in the “TSdist” R package [30]. Shape Based Distances [31] were calculated using SBD as implemented in the “dtwclust” package [32]. Nearest neighbor clustering was performed with hclust and nearest neighbor classification was performed using the package “class” [33]. Silhouette values [34] were calculated using the “cluster” package [35].

Graphics were produced using ggplot2 [36] and ggdendro [37].

### DNA sequencing

Species identity was determined by nucleotide sequence comparison when high-resolution melt predictions and biochemical determinations were discordant. The ITS region was PCR amplified with primers NSI1 (GATTGAATGGCTTAGTGAGG) and NLB4 (GGATTCTCACCCTCTATGAC) [38], except for *C*. *lusitaniae*, which lacks the NLB4 target site. ITS4 was used in place of NLB4. The size of the PCR product and specificity of the reaction was determined by agarose gel electrophoresis and both strands of the amplified DNA were sequenced (Genewiz) using each of the amplification primers. Sequences were analyzed using DNA Strider [39] and sequence comparisons against the Genbank non-redundant database were performed using BLAST [40]. Sequence alignments were performed with Clustal Omega [41].

## Results

### Choice of amplicon

The objective of these studies was to assess the utility of distance methods developed for comparison of time series data to classify high-resolution melt (HRM) curves as a means of fungal species identification. Rather than rely on a single parameter such as melting point, this approach takes into consideration the entire melt curve and thus incorporates significantly more information, which should generate a more robust and discriminating measure [42]. It also facilitates objective evaluation in an automated manner.

The ITS region was chosen as the target amplicon for several reasons. The Fungal Barcode Consortium recently proposed the ITS region as the universal barcode for fungal species based on the level of sequence variability and utility in discriminating species, as well as its multi-copy nature and high rate of successful amplification [43]. In addition, a number of studies have focused on the design of pan-fungal primers. In this study the primers proposed by Toju et al. [24], which overcome some of the bias documented in other pan-fungal primers [44], were used. Furthermore, HRM studies targeting this region have demonstrated species variability in melt profiles [5–10].

**Fig 1:**
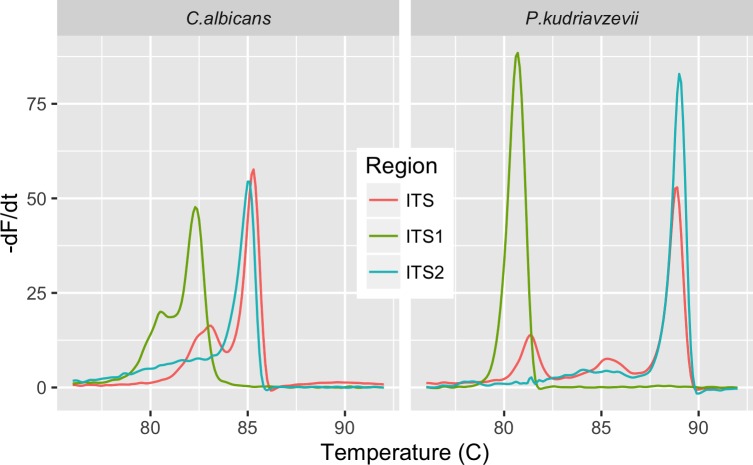
Variation in HRM profile of ITS regions. The negative first derivative (-dF/dt) of the normalized melt curve of rDNA ITS1, ITS2 and full-length ITS region of *C*. *albicans* and *P*. *kudriavzevii* are shown.

While prior studies have examined HRM profiles of various amplicons derived from the ITS region, no study has compared the melt profiles of the ITS1, ITS2, and composite ITS region to assess which might prove most discriminating. DNA melting is a function of base composition and sequence, which establishes the number, size, and relative position of cooperative melt domains [4]. Therefore, the melt profile of the composite ITS region is not the simple sum of ITS1 and ITS2 curves. Two examples of this distinction are shown in Fig 1. As pointed out by Rasmussen et al. [45], a more complex, multimodal pattern facilitates species discrimination. Therefore, the melt profiles of ITS1, ITS2, and full-length ITS region were examined to assess which generated the most diverse HRM curves.

**Fig 2:**
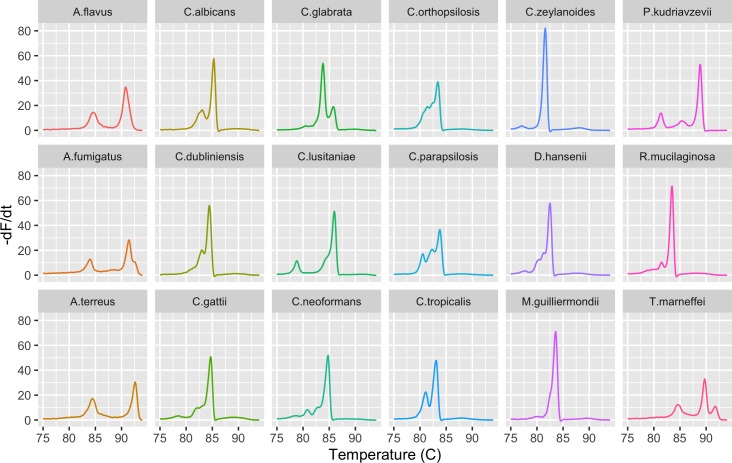
The melt profile of the ITS region is unique to each species. The negative first derivative (-dF/dt) of the normalized melt curve of the ITS region is shown for a representative strain of each of the species listed in Table 1.

Fifty-one strains encompassing 18 species (Table 1) were examined. These included yeast and molds, Ascomycetes and Basidiomycetes; most are important human pathogens. The most diverse set of melt profiles was observed for the full-length ITS region (Fig 2, S1 and S2 Figs). The curves varied in number of peaks, peak heights, and peak position, resulting in a unique profile for each species. All but one profile, that of *M*. *guilliermondii*, had at least two distinct peaks. The profiles from even closely related species, such as *Candida parapsilosis* and *Candida orthopsilosis*, *Candida albicans* and *Candida dubliniensis*, *Cryptococcus neoformans* and *Cryptococcus gattii*, were easily distinguished by visual examination. In contrast, the melt profiles of the ITS1 and ITS2 regions were more often a single peak curve and the distinction between some species was not readily apparent (S1 and S2 Figs).

**Fig 3:**
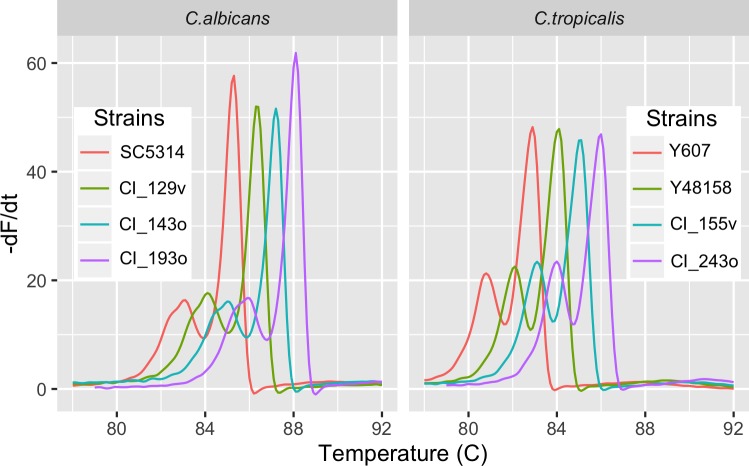
The melt profile is conserved across different strains of the same species. The negative first derivative (-dF/dt) of the normalized melt curve of the ITS region is shown for four strains of the *C*. *albicans* and *C*. *tropicalis*. Each successive curve is offset by +1°C to facilitate comparison of the curve shapes.

At least two strains of each species were examined as an initial gauge of the constancy of curves within a species. As shown in the representative examples of *C*. *albicans* and *C*. *tropicalis* (Fig 3), each was characterized by a highly similar and reproducible curve, as were all the other species. As evident later, this is not always true as the constancy of the melt curves depends on the number and location of ITS sequence variations within a species.

### Dynamic time warping as a distance measure

**Fig 4:**
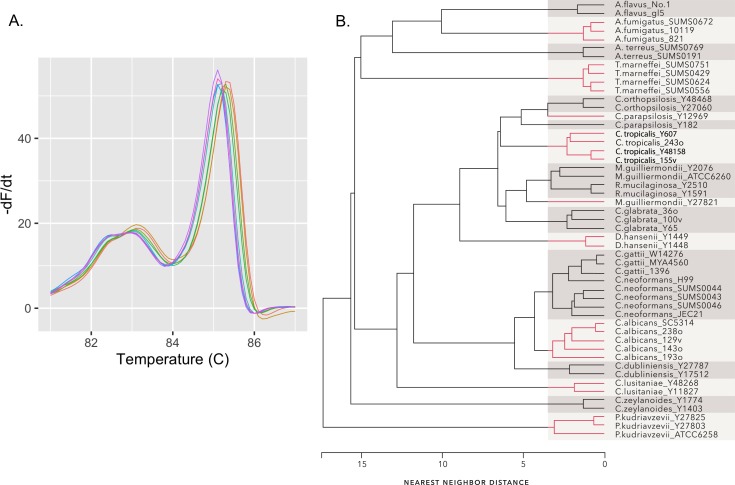
Euclidean distance is a poor metric for comparing melt curves. (A) Melt profiles of eight samples of *C*. *albicans* strain SC5314 illustrating the variation inherent in melt curve acquisition. (B) Dendrogram of nearest neighbor clustering results using Euclidean distances. The four melt curves obtained for each strain in Table 1 were averaged and the Euclidean distances between the averaged curves was clustered.

One of the inherent problems in HRM is the run-to-run differences due to sample and machine variables [46] (Fig 4A). This variability confounds inelastic measures such as Euclidean or Manhattan distances. As an example, Fig 4B shows a cluster dendrogram constructed using Euclidean distances between the average curve of each of the 18 test species. When cut at a tree height that produces 18 groups, a number of the species are correctly clustered, but several are not. In particular, *C*. *neoformans* and *C*. *gattii* are not separated, nor are *M*. *guilliermondii* and *R*. *mucilaginosa*. Parenthetically, it should be noted that although these cluster dendrograms mimic the appearance of phylogenetic dendrograms, they do not reflect nor imply phylogenetic relationships.

In contrast, DTW was introduced to compensate for temporal distortions [21] analogous to the temperature distortions seen in HRM data. The DTW function is governed by a local step pattern and a global window constraint [21]. Giorgino’s R implementation of the DTW algorithm [19] includes all 43 step patterns defined by Rabiner and Juang [21]. These consist of seven types [1–7] with four possible slopes (a,b,c, and d), as well as smoothed variants of 15 of the basic 28 patterns. There is no theoretical basis for selection of a particular step pattern and their utility must be empirically assessed in the context of the data type being evaluated [21]. The 43 available step patterns were tested against a data set consisting of 204 melt curves generated from the 51 strains in Table 1, two independent duplicate curves from each. Inclusion of the four curves from each strain helped ensure that the method was robust against technical variations. A distance matrix was calculated using each of the possible step patterns, either without window constraints or with a window size of 1 to 20, corresponding to a temperature range of 0.1°C to 2°C.

**Fig 5:**
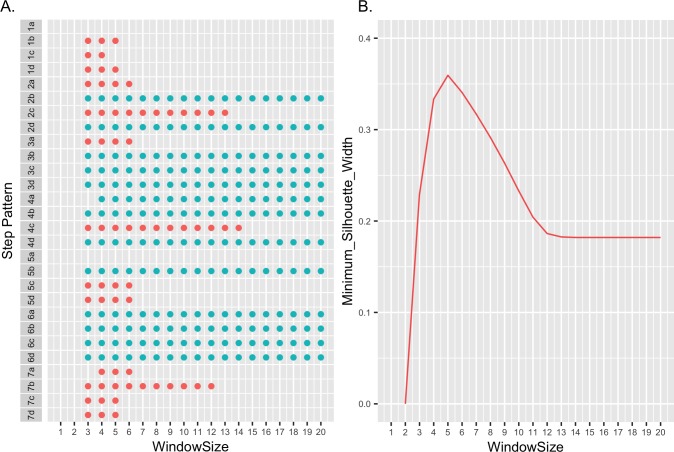
The effect of DTW step patterns and window sizes on melt curve clustering. (A) DTW distances between all 204 melt curves obtained for the 51 strains in Table 1 were calculated using each of the possible Rabiner and Juang step patterns and slope combinations (21). Distances were calculated with no window constraint or with the window size varied from 1 to 20. Dots indicate successful nearest-neighbor clustering of all 204 melt curves into 18 species-specific groups. Green dots indicate step pattern and slope combinations successful even in the absence of window constraints. Red dots represent those distances for which clustering was successful only with the indicated window size. (B) The minimum silhouette width (34) as a function of window size for step pattern Type 6b.

The effectiveness of each step pattern was assessed using one-nearest-neighbor classification with stratified eight-fold cross-validation [20]. With the exception of the Type Ia step pattern, which had an accuracy rate of <0.3, the other step patterns performed equally well, having accuracy rates of >0.99. The distance measures were further evaluated by nearest-neighbor clustering [47, 48] under the assumption that distances most reflective of the unique curve shapes would allow clustering into eighteen distinct species-related groups. As shown in Fig 5A, distances computed by 13 of the step patterns resulted in accurate clustering of all the curves in the absence of any window constraint. Three general themes emerged. The Type 6 step pattern was effective regardless of the slope, the most effective slope was “b”, which was effective with five of the seven step pattern types, and step pattern Types 1 and 7 were ineffectual. When globally constrained, effective clustering was obtained with distances computed with all possible step patterns except Type 1a and 5a (Fig 5A). However, the size of the window constraint required for successful clustering varied (Fig 5A). The smoothed patterns showed little or no difference.

**Fig 6:**
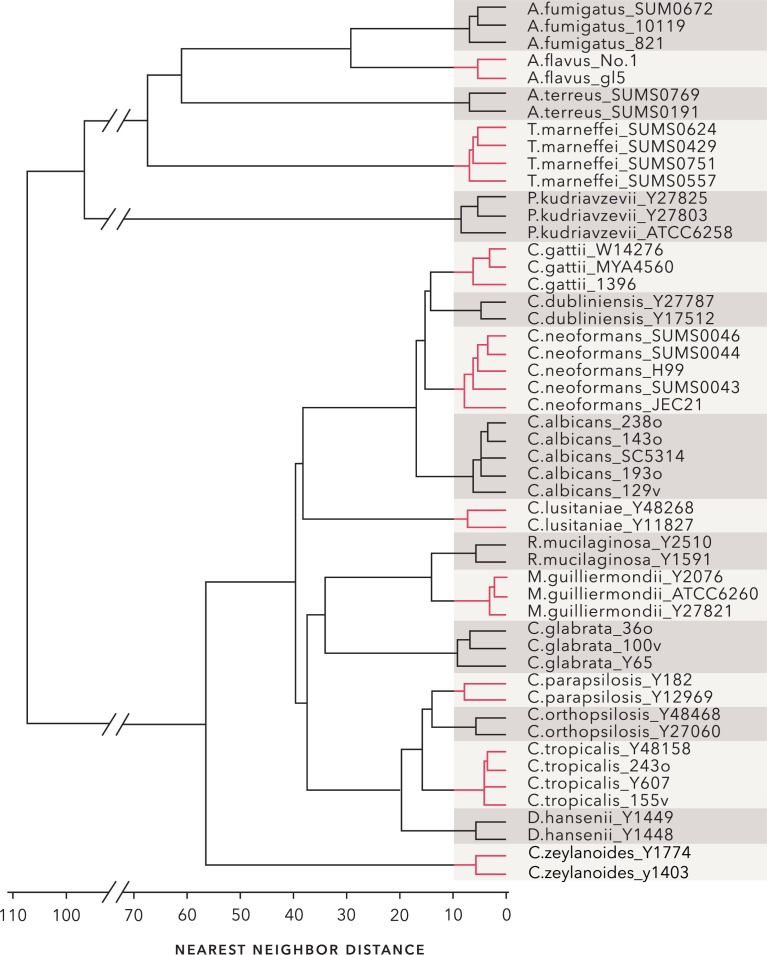
Nearest-neighbor clusters formed from DTW distances. DTW distances between the average melt profile from each of the 51 strains in Table 1 were calculated with step pattern Type 6b and a window size of five. The dendrogram resulting from nearest neighbor clustering of the distances is shown. The tree can be cleanly cut into 18 groups, each corresponding to a different species.

Based on these results a Type 6 step pattern with a slope of “b” was chosen for subsequent analyses. The optimum window size for the Type 6b pattern was evaluated by examining the minimum silhouette width as a function of window size. The minimum silhouette width provides a relative measure of the distance between members of a cluster and the next closest cluster [34]. As seen in Fig 5B, the minimum silhouette width increases dramatically as the window size decreases below 12 and reaches an optimum at a value of 5. The dendrogram in Fig 6 illustrates clustering of distances between the average curve of each strain computed with a Type 6b step pattern and a window size of 5. The dendrogram is easily cut into 18 well-separated groups each corresponding to a different species.

### Classification of clinical isolates using DTW distances

The foregoing demonstrated that DTW distances are a useful metric for discriminating HRM profiles and concomitantly the corresponding fungal species. The utility of DTW distances for classification of unknown samples was evaluated in a blinded test of a collection of previously identified oral and vaginal yeast isolates from HIV-infected and non-infected women [22]. A reference database of standard curves was prepared for each of the yeast strains in Table 1. Two independent duplicate melt curves were obtained for each strain and averaged to produce the reference standard. Duplicate HRM profiles were acquired for each clinical isolate. For both the reference strains and clinical strains, a rapid freeze/thaw method [8] was used to prepare a crude DNA sample. The same method of DNA preparation was used for both sets of strains to control for the potential influence of other cellular components on the melt profiles. A total of 251 strains including eight positive controls and 243 clinical isolates were evaluated. The DTW distance between the duplicate curves of each clinical isolate and each of the database standards was determined with a Type 6b step pattern and a window size of 5.

Classification of the test isolates was based on minimum distance matching against the database standards. That is, the standard curve least distant from the test curve was considered the match. Results were scored as a valid identification when both duplicate test curves matched the same species in the database and both distances were at or below the cutoff value. The cutoff score was established by determining the mean within-strain distance of the reference curves and their standard deviation. The within-strain distance is the distance between each of the four curves obtained for each strain and provides the range of distance values to be expected for ostensibly identical curves. The cutoff value was set at the mean +2 standard deviations of the within-strain distance. A probable identification resulted when both curves matched the same species, but only one score lay below the cutoff value. When both distance scores exceeded the cutoff, the sample was classified as “unidentified.” Samples scored as probable or unidentified were tested again. The distance-based classifications were then compared to those previously established by phenotypic and biochemical methods [22].

A valid identification was obtained for all eight positive controls, and all were classified as the correct species. These included four strains of *C*. *albicans*, two of *C*. *glabrata*, and two of *C*. *tropicalis*. Of the 243 test strains a valid ID was obtained for 195 (Table 2). For all but three of these 195 strains, the DTW distance-based classification agreed with the previously determined identification. One isolate, previously identified as *C*. *lusitaniae*, was classified as *C*. *tropicalis* based on the melt profile and this was confirmed by nucleotide sequence analysis of the ITS amplicon. The sequence was 100% identical to *C*. *tropicalis* isolate 287 (Genbank Acc.# KU950724) [49]. An isolate previously identified as *D*. *hansenii*, was determined to be *C*. *lusitaniae* based on the melt profile and ITS sequence, which was 100% identical to *C*. *lusitaniae* ATCC34449 (Genbank accession # KU729100). The third discordant identification was a strain of *C*. *parapsilosis* that proved to be *C*. *orthopsilosis* (ITS sequence identical to *C*. *orthopsilosis* ATCC96141, Acc.# EU564208). The last is not surprising as *C*. *parapsilosis* and *C*. *orthopsilosis* are not easily distinguished by phenotypic means [50] and this illustrates the ability of DTW distance measures to discriminate species that are difficult to identify by routine biochemical tests.

**Table 2:** Identification results for 243 clinical yeast isolates.

Species	Identification Method				Comments
	Phenotypic	DTW	EDR	SBD	
*C*. *albicans*	191	145	145	144 (1)*	
*C*. *boidinii*	1	UnID	UnID	UnID	
*C*. *dubliniensis*	-	46 (2)	46	45 (2)	all originally classified as *C*. *albicans*
*C*. *glabrata*	26	26	26	26	
*C*. *lusitaniae*	2	2	2	2	one isolate identified as *C*. *tropicalis*
*C*. *orthopsilosis*	-	1	1	1	
*C*. *parapsilosis*	8	7	7	7	one isolate identified as *C*. *orthopsilosis*
*C*. *tropicalis*	8	9	9	9	
*C*. *zeylanoides*	1	1	1	1	
*D*. *hansenii*	1	0	0	0	identified as *C*. *lusitaniae*
*Kloeckera spp*.	1	UnID	UnID	UnID	
*P*. *kudriavzevii*	1	1	1	1	
*R*. *mucilaginosa*	3	3	2	2	one isolate unidentified by EDR and SBD

* numbers in parenthesis indicate “probable” identifications.

UnID—unidentified.

Forty-eight test strains were scored as “unidentified”. Two of these corresponded to isolates previously identified as *Candida boidinii* and a *Kloeckera spp*. Since the database did not contain standard curves for either of these species, the isolates were correctly classified as unidentified. They were true negatives.

**Fig 7:**
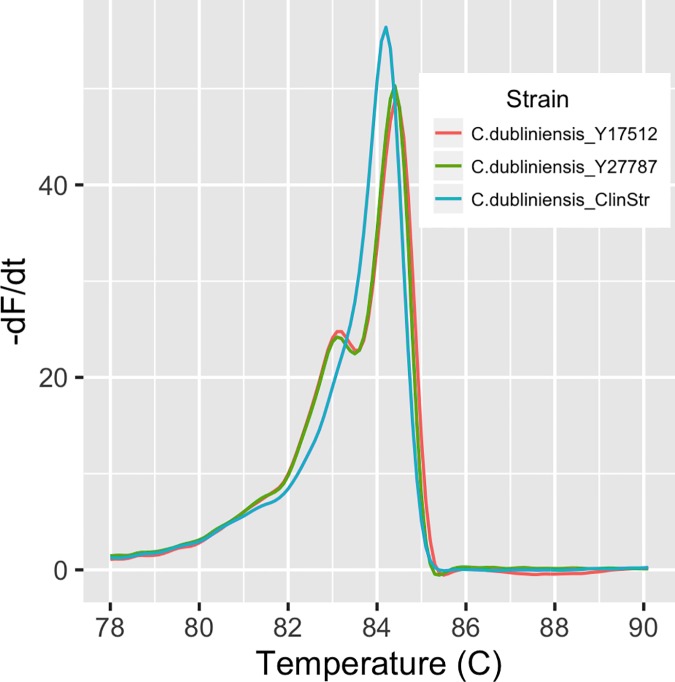
Melt profiles differ between *C*. *dubliniensis* subtypes. The melt profile of a *C*. *dubliniensis* Genotype 1 strain, clinical isolate CI_39o, and the two database standards, both of Genotype 2, is shown.

The other 46 unidentified strains were interesting in that they had ostensibly identical melt profiles, which differed from those in the database. All were oral isolates previously identified as *C*. *albicans*, 35 isolated from 15 HIV positive women and 11 isolated from 4 HIV negative patients [22]. The ITS region of 19 of these strains was sequenced to determine their identity. All 19 sequences were identical to each other and 100% identical to *Candida dubliniensis* strain *CD36* (Acc. # FM992695.1), for which the entire genome sequence is available [51]. This was surprising since our reference database contained curves for two *C*. *dubliniensis* strains. Although the melt profile of the *C*. *dubliniensis* database strains overlapped that of the oral isolates, the profiles clearly differed in shape (Fig 7). Comparison of ITS nucleotide sequences showed that the database strains were indeed *C*. *dubliniensis*, but their sequence differed from that of the oral isolates (S3 Fig). The oral isolates belong to Genotype 1, as defined by Gee et al. [52], whereas the strains used as standards belong to Genotype 2. This sequence difference presumably accounts for the distinct melt profiles. When the database was updated by inclusion of a Genotype 1 curve, reanalysis of the clinical isolates resulted in classification of each of the 46 oral isolates as *C*. *dubliniensis*.

*C*. *albicans* and *C*. *dubliniensis* are closely related and are distinguished by specialized tests not routinely performed in clinical labs [53]. The prior study utilized only routing tests [22] and thus it is not surprising that these 46 strains were misidentified as *C*. *albicans*. Importantly, the DTW distance classification demonstrates again the capacity of this method to distinguish species that are difficult to identify by phenotypic and biochemical means and, furthermore, illustrates the potential to discriminate even within a species.

The database encompassed all but two species found amongst the clinical isolates. These two isolates were correctly classified as unidentified, as were the Genotype I *C*. *dubliniensis* isolates prior to incorporation of an appropriate standard. This suggested that the method is robust against false positive identifications. As a further test of this, the clinical isolates were reanalyzed against a database lacking one set of species standards. The absence of each species was tested. In each trial the unidentified clinical isolates corresponded to the missing database species and no false positive classifications occurred.

### Alternative distance measures

While DTW is a widely used distance measure, a number of other distance functions have been described that approach DTW in classification accuracy and at reduced computational cost [20]. These include Edit Distance on Real sequence (EDR) [27], Edit distance with Real Penalty (ERP) [28], and Longest Common Subsequences (LCSS) [29]. As done with DTW, each of these functions was used to evaluate distances between the melt profiles of the all the strains in Table 1, and the utility of the distance measures was assessed by 1NN classification and nearest neighbor clustering.

**Fig 8:**
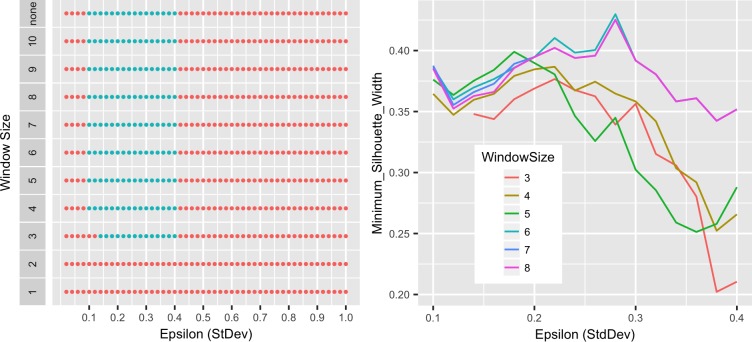
Optimization of EDR distance parameters. (A) EDR distances were calculated with the indicated epsilon values and window constraints. Green dots represent distance values resulting in successful nearest-neighbor clustering of all 204 melt curves in 18 species-specific groups. Red dots represent those parameter values for which clustering failed. (B) The minimum silhouette width (34) as a function of window size was determined for each epsilon value associated with successful clustering.

**Fig 9:**
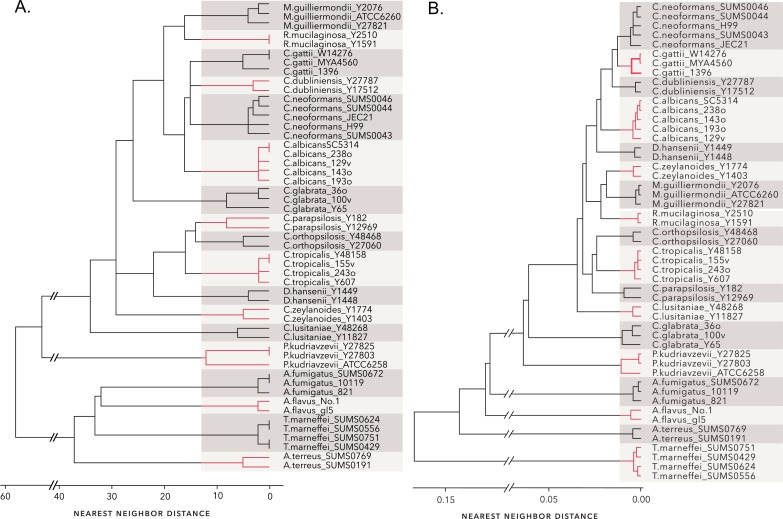
Nearest-neighbor clusters formed from EDR and SBD distances. (A) EDR distances between the average melt profile from each of the 51 strains in Table 1 were calculated with an epsilon value of 0.28 standard deviations and window size of 10. The dendrogram resulting from nearest neighbor clustering of the distances is shown. (B) The dendrogram resulting from nearest neighbor clustering of SBD distances is shown. Both trees can be cut into 18 groups, each corresponding to a different species.

EDR distances proved equally useful. EDR requires tuning of the parameter ε, the threshold difference at which two data points are considered the same or different [27]. As with DTW, EDR also allows for a window constraint [27]. As suggested by Ding et al. [20], epsilon values ranging from 0.02 standard deviations of the data set to 1 standard deviation were tested in 0.02 increments. Additionally, the function was tested with no window constraint or window sizes of 1 to 10. 1NN classification indicated that ε values between 0.04 and 0.68 standard deviations were acceptable, however, cluster results suggested a narrower range of ε values to be optimal, 0.1 to 0.4 standard deviations (Fig 8A). This was not significantly affected by window size, but comparison of minimum silhouette widths indicated that window sizes less than six resulted in poorer clustering and that minimum silhouette widths were optimal with an ε value of 0.28 standard deviations (Fig 8B). An example of the clustering of EDR distances is shown in Fig 9A. As with DTW distances, the dendrogram could be cut into 18 distinct species-related groups.

The accuracy of EDR classification of unknowns was tested in the same manner as for DTW distances. The same set of database standards and clinical isolate curves were compared using EDR distances computed with an epsilon value of 0.28 standard deviations and a window size of 10. As shown in Table 2, EDR distances functioned nearly as well as DTW distances. Like DTW, EDR correctly identified the positive control strains and 194 test isolates, including the miss-identified strains of *C*. *lusitaniae*, *C*. *orthopsilosis*, and *C*. *tropicalis*. However, EDR failed to identify one isolate of *R*. *mucilaginosa*. The same 48 test strains classified as unidentified by DTW were also classified as unidentified by EDR and inclusion of the additional *C*. *dubliniensis* standard in the database allowed identification of all 46 *C*. *dubliniensis* isolates.

A recently introduced distance measure, “Shape-based Distance” (SBD), a normalized cross-correlation measure [31] also functioned well. SBD has no optimizable parameters and was tested directly. Nearest neighbor classification was 100% accurate using the SBD distances and nearest neighbor clustering resulted in 18 distinct species-related clusters (Fig 9B). When used to classify the clinical isolates, its performance was also comparable to DTW, but, in addition to its failure to identify one isolate of *R*. *mucilaginosa*, one *C*. *dubliniensis* isolate was also classified as “unidentified.”

Lastly, LCSS and ERP were also tested, but distances generated by both functions failed to yield accurate clusters.

## Discussion

High-resolution melt analysis is an attractive method of assessing nucleotide sequence differences because of its low cost, rapidity, and simplicity. However, sample and machine variables limit reproducibility and complicate comparison of melt curves [46]. This variability is commonly dealt with in a limited way by temperature shifting, the alignment of similar curves based on input from the investigator [3] or statistical grouping of similar curves [54]. An alternative approach has been the incorporation of internal standards with melting points that flank the curves of interest and against which the test curves can be adjusted [3]. Aside from the time and labor required by such semi-automated methods, their application to identification of unknown samples is problematic. Temperature shifting requires either prior knowledge that the curves are similar or an a priori assumption of similarity. When analyzing unknown samples, prior knowledge is not available and similarity cannot be assumed. Internal standards also require prior knowledge and it is unlikely a standard could be defined that flanks the high Tm of the curves seen with the ITS region of *Aspergillus spp*. (Fig 2).

DTW was introduced to identify similar curves despite temporal distortion [21]. It has broad applicability to curves generated by time series data or other types of serial data [19, 20, 48] and, as shown here, proved equally useful in the analysis of temperature series data. Distance determinations in DTW are controlled globally by window size constraints and locally by step patterns and slopes [19]. Screening of these parameters showed the algorithm to be rather flexible. Discriminating distances, as judged by nearest neighbor clustering, were produced with varied combinations of local and global constraints, although some step patterns, types 3, 4 and 6, were generally more successful. This apparent flexibility may be related to the limited data set examined and defining an optimum set of parameters will require examination of larger data sets. Regardless of whether there exists a single optimum set of parameters for melt curve analysis, it is clear that the algorithm deals effectively with the run-to-run variability seen with HRM [46].

Applying distance measures to HRM curves for fungal classification requires that the HRM curves have sufficient diversity and complexity to distinguish all the species of interest. The ITS region was an attractive target in light of its adoption as the universal DNA barcode for fungal classification [43]. While prior HRM studies had focused on this region, there had been no systematic examination of curve shapes associated with various amplicons from the ITS region. Examination of the ITS1, ITS2 and composite ITS1-5.8S-ITS2 HRM profiles of 18 fungal species showed that the composite ITS amplicon exhibited a greater complexity and range of shapes than either ITS1 or ITS2 and that a unique melt curve was associated with each species. DTW distances effectively distinguished each species-specific curve as demonstrated by nearest-neighbor classification and clustering.

The value of DTW-based classification was demonstrated by its ability to correctly identify over 200 clinical isolates. Using a minimum distance scoring method, 241 of the 243 tested isolates were correctly identified. Two of the isolates were classified as “unidentified,” and, since corresponding reference curves were absent from the database of standard curves, these were true negative results. Furthermore, the outcome exposed 49 misidentified isolates. Two were clear misidentifications, or perhaps mislabeled samples. The distance method correctly identified strains of *C*. *tropicalis* and *C*. *lusitaniae* previously identified as *C*. *lusitaniae* and *D*. *hansenii*, respectively. Of more interest, distance matching identified as *C*. *orthopsilosis* a strain previously typed as *C*. *parapsilosis*. These are closely related strains that are difficult to distinguish by traditional phenotypic schemes [50]. Similarly, 46 isolates, initially scored as unidentified, proved to be *C*. *dubliniensis*, but different in genotype from the *C*. *dubliniensis* strains used as database standards. These isolates were previously identified as *C*. *albicans*. Distinguishing *C*. *dubliniensis* from *C*. *albicans* requires phenotypic tests that are not routinely performed in the clinical lab [53] and misclassification of *C*. *dubliniensis* is a common problem.

The unique melt curves associated with the two *C*. *dubliniensis* genotypes has both positive and negative implications. While it illustrates the capacity of HRM curve classification to discriminate species subtypes, it also indicates that a useful database of standard curves will need to incorporate multiple curves to define some species. The number of representative curves will depend on the genetic diversity within a given species and the effect of specific nucleotide differences on the melt curve. Interestingly, nucleotide diversity within the ITS regions is much greater for *C*. *albicans*, *C*. *glabrata*, and *C*. *tropicalis* than for *C*. *dubliniensis* [55]. Yet, curve matching correctly identified 145 clinical isolates of *C*. *albicans*, 26 of *C*. *glabrata*, and 9 of *C*. *tropicalis*. Also, the two strains of *C*. *dubliniensis* initially used as database standards were not identical in nucleotide sequence, but had indistinguishable melt profiles. This suggests that subtype curve variance may be a limited problem and highlights the point that melt curves are more critically influenced by the type and position of nucleotide differences rather than the mere number of differences [4]. It also suggests that database construction must be a largely empirical process, unless predictive algorithms can identify nucleotide differences with the potential to influence the shape of the melt curves. Several algorithms have been described for prediction of melt curves, including POLAND [56], MELTSIM [4] and uMELT [57] but, neither POLAND or uMelt successfully predict the distinction in *C*. *dubliniensis* melt curves. However, it is not possible to enter accurate parameters into these algorithms because of the proprietary nature of HRM mixes and the algorithms do not account for the thermodynamics of dye binding.

Examination of eighteen fungal species, including many of the most clinically relevant species, showed that each had a distinctly different melt curve and that DTW distances were a useful metric for their identification. However, this is a limited sample of fungal species and the question remains as to how extensive a variation in melt curves will be seen as more species are examined and how robust DTW will be in distinguishing subtle differences in curve shape. It is possible that the melt profile of the ITS region will be inadequate and additional information may be needed. In this regard it should be noted that the melt profiles of the ITS1 and ITS2 regions, which differ substantially from that of the composite ITS region, might be added to the analysis, similar to the 2-D Tm analysis of Bergman et al. [58].

In addition to DTW, a number of other distance functions have been developed for time series analysis [20]. Both EDR and SBD distances effectively distinguished all the melt curves and were very close in performance to DTW for classifying unknown samples (Table 2). Analysis of a larger dataset will be needed to determine which function provides optimal resolution. It will also be of interest to compare these with other classification methods that have been applied to melt curve analysis such as support vector machines [59].

The approach outlined in these studies could significantly improve the time, effort, and accuracy of identifying cultured samples. Although the method was tested against clinical isolates of yeast only, it should readily extend to filamentous species as well since these were easily classified among the standard curves. Ideally, identification of fungal pathogens directly from clinical specimens would substantially reduce time to diagnosis. This goal introduces a number of ancillary issues such as methods of sample processing and DNA extraction, PCR amplification efficiency and primer specificity. Pan-fungal primers as used in this study, of necessity, target conserved regions of the rDNA potentially leading to interference from host DNA in clinical samples. However, there are adequate differences between fungal and human rDNA to allow fungal-specific primer design. The occurrence of mixed infections is also potentially problematic, but might be addressed by computational curve resolution methods [60].

## Supporting information

S1 Fig Melt profiles of the ITS1 region of various species. The negative first derivative (-dF/dt) of the normalized melt curve of the ITS1 region is show for a representative strain of each of the species listed in Table 1.
(TIF)

S2 Fig Melt profiles of the ITS2 region of various species. The negative first derivative (-dF/dt) of the normalized melt curve of the ITS2 region is show for a representative strain of each of the species listed in Table 1.
(TIF)

S3 Fig Comparison of *C*. *dubliniensis* ITS nucleotide sequences. The ITS nucleotide sequence of the Genotype 1 strain, clinical isolate CI_39o, and Genotype 2 strains, Y17512 and Y27787, were aligned with Clustal Omega (41). Differences between Genotype 1 and 2 are highlighted in red. The single nucleotide difference between the Genotype 2 strains is highlighted in green.
(TIF)
