# Supplementary figures and images for: Dynamic time warping assessment of high-resolution melt curves provides a robust metric for fungal identification
Source: PLoS One. 2017 Mar 6;12(3):e0173320. doi: 10.1371/journal.pone.0173320 (PMC5338801; doi:10.1371/journal.pone.0173320)

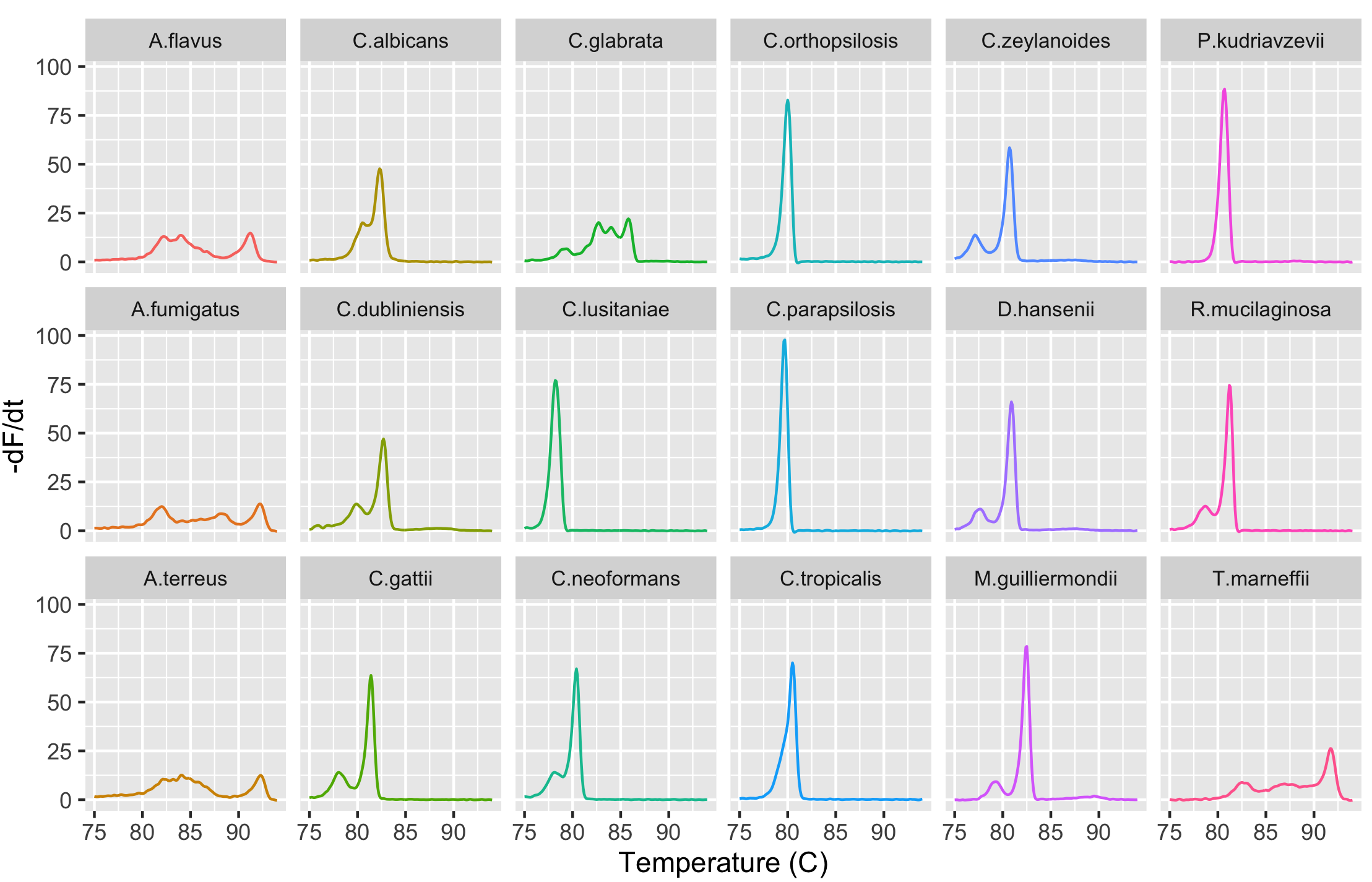

Supplement: S1 Fig — The negative first derivative (-dF/dt) of the normalized melt curve of the ITS1 region is show for a representative strain of each of the species listed in Table 1. (TIF) [file pone.0173320.s001.tif]

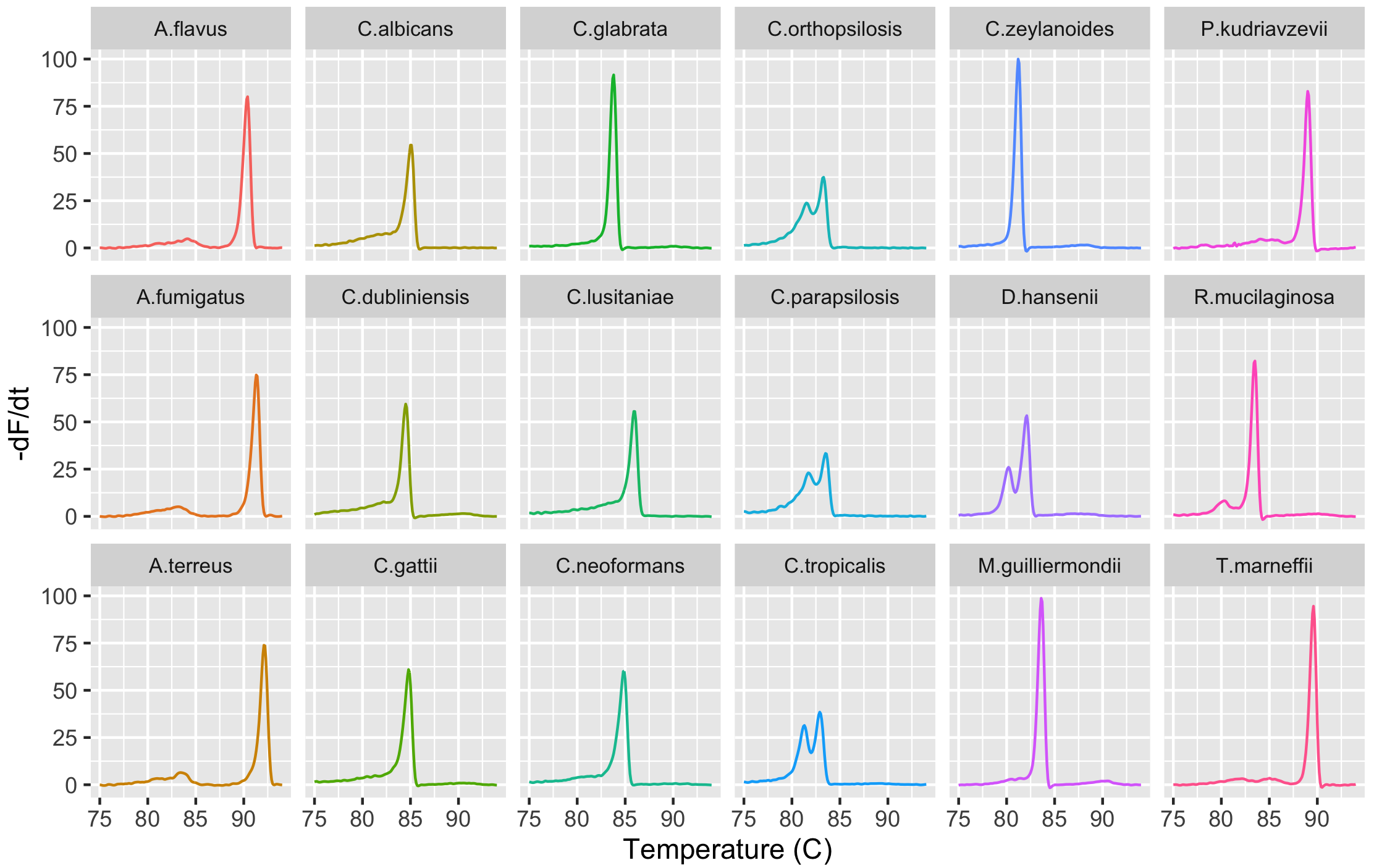

Supplement: S2 Fig — The negative first derivative (-dF/dt) of the normalized melt curve of the ITS2 region is show for a representative strain of each of the species listed in Table 1. (TIF) [file pone.0173320.s002.tif]

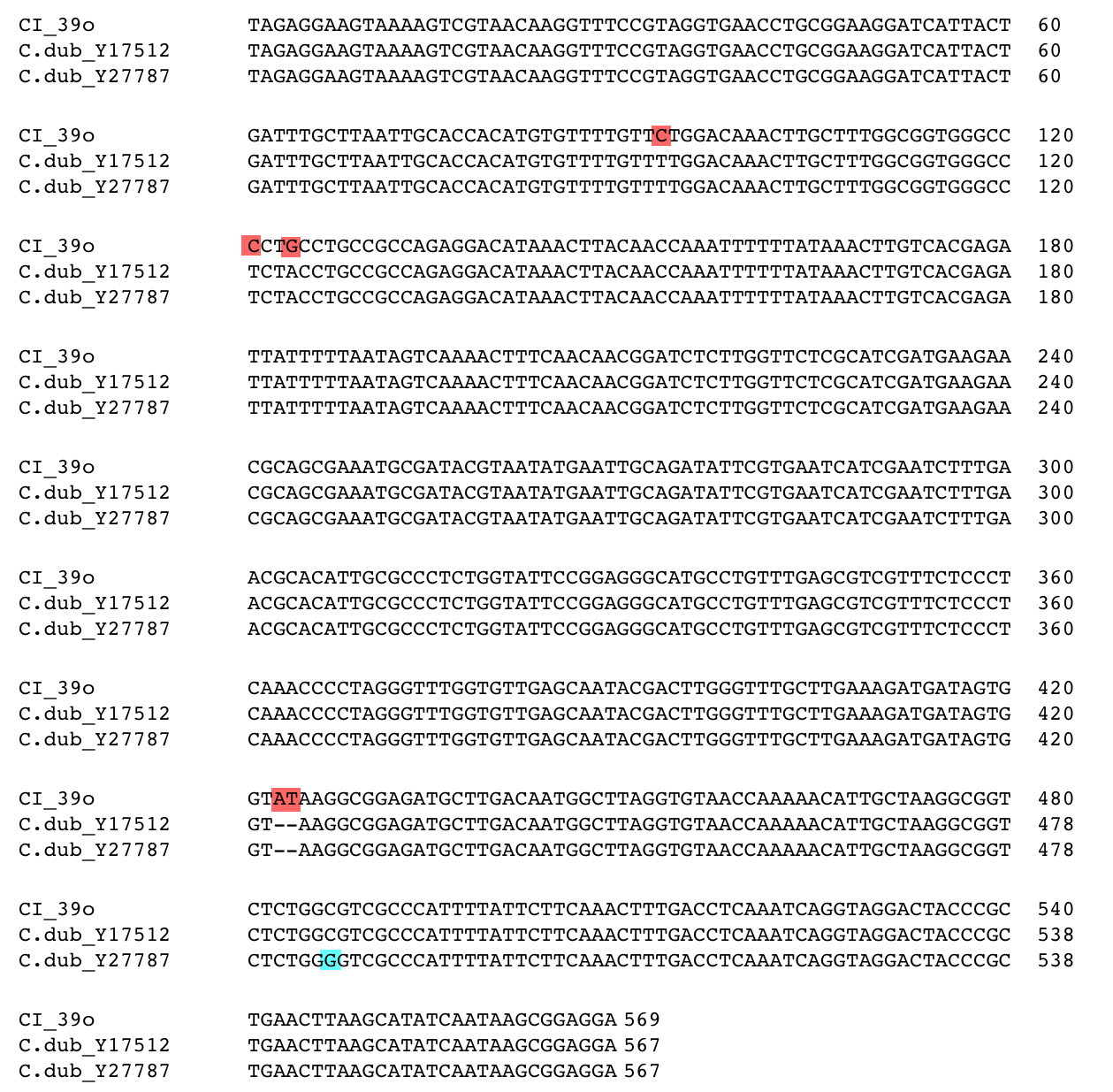

Supplement: S3 Fig — The ITS nucleotide sequence of the Genotype 1 strain, clinical isolate CI_39o, and Genotype 2 strains, Y17512 and Y27787, were aligned with Clustal Omega (41). Differences between Genotype 1 and 2 are highlighted in red. The single nucleotide difference between the Genotype 2 strains is highlighted in green. (TIF) [file pone.0173320.s003.tif]
